# Understanding selective predation: Are energy and nutrients important?

**DOI:** 10.1371/journal.pone.0201300

**Published:** 2018-08-08

**Authors:** Tamara I. Potter, Hayley J. Stannard, Aaron C. Greenville, Christopher R. Dickman

**Affiliations:** 1 Desert Ecology Research Group, School of Life and Environmental Sciences, University of Sydney, Sydney, New South Wales, Australia; 2 Marsupial Nutrition Research, School of Life and Environmental Sciences, University of Sydney, Sydney, New South Wales, Australia; 3 National Environmental Science Programme Threatened Species Recovery Hub, University of Sydney, Sydney, Australia; 4 Long Term Ecological Research Network, Terrestrial Ecosystem Research Network, University of Sydney, Sydney, Australia; Ben-Gurion University of the Negev, ISRAEL

## Abstract

For generalist predators, a mixed diet can be advantageous as it allows individuals to exploit a potentially broad range of profitable food types. Despite this, some generalist predators show preferences for certain types of food and may forage selectively in places or at times when these foods are available. One such species is the lesser hairy-footed dunnart (*Sminthopsis youngsoni*). Usually considered to be a generalist insectivore, in the Simpson Desert, Australia, this small marsupial predator has been found to selectively consume wolf spiders (Family Lycosidae), for reasons yet unknown. Here, we tested whether lycosids have relatively high energy or nutrient contents compared to other invertebrates, and hence whether these aspects of food quality can explain selective predation of lycosids by *S*. *youngsoni*. Energy, lipid and protein composition of representatives of 9 arthropod families that are eaten by *S*. *youngsoni* in the Simpson Desert were ascertained using microbomb calorimetry, chloroform-methanol extraction and Dumas combustion, respectively. Although lycosids contained a high proportion of energy and nutrients, they were not found to yield statistically greater amounts of these food components than many other available arthropod prey that are not selected by *S*. *youngsoni*. Our results therefore suggest that alternative factors may be more influential in shaping dietary selection in this marsupial predator, such as high rates of encounter between lycosids and *S*. *youngsoni*.

## Introduction

When animals forage, a trade-off exists between maximizing net energy and nutritional gain, and minimizing costs such as predation, time and energy expenditure [1–3]. According to optimal foraging theory, predators should therefore prefer foods that deliver the greatest value per unit of effort or time expended acquiring them [4–6].

When the most profitable food type is constant, some predators develop morphological or physiological specialisations that constrain them to that prey (e.g., reduviids, or assassin bugs, have piercing, tubular mouthparts that restrict them to feeding on the coelomic fluid of other arthropods), but many others are generalists that have mixed diets [6–8]. For these, search, capture and handling costs, as well as the nutritional composition of prey fluctuate spatially, seasonally, taxonomically and with ontogeny [6, 7, 9]. Thus, the capacity to consume a wide variety of food types may have significant advantages for survival and persistence. Despite this, predators across a wide array of taxa, including invertebrates [10–12], fish [13, 14], and all classes of terrestrial vertebrates [15–19], have been documented to complement their mixed diet through selective predation on a restricted range of prey [7].

Traditionally, energy has been considered to be the primary currency motivating foraging decisions [2, 20]. However, there is increasing evidence that the need for specific nutrients also influences when, where and for how long individuals forage [9, 10, 20, 21]. As the proportion of certain macronutrients (i.e., protein, lipid and carbohydrates) consumed by animals is often associated directly with fitness attributes such as longevity, fecundity, growth and body size [22–24], there is a strong incentive to forage selectively to reach a particular nutrient intake target [7]. For example, a study on the diet of herring gulls (*Larus argentatus*) suggested that energy *per se* is not the only food component that governs fitness in the wild, as gulls that specialized on mussels achieved higher reproductive output than more generalist-feeding gulls, even though mussels were the least energy-dense prey available [7, 25]. Additionally, research into prey selection in two species of insectivorous marsupials found a preference for prey body parts with a high lipid content [7, 26].

This study explores the composition of different types of prey in the diet of an Australian desert-dwelling insectivore, the lesser hairy-footed dunnart (*Sminthopsis youngsoni*). Although considered to be a generalist that hunts a diverse range of arthropods [27, 28], a recent study suggested that wolf spiders (Family: Lycosidae) are consumed disproportionately often relative to their availability and that of other spiders [18]. Thus, lycosids formed 53% of the spider component of the diet of *S*. *youngsoni*, as determined by examination of scats, but only 18% of the spiders—from 14 families—that were available in the foraging habitats of the predator [18]. Confirmation that *S*. *youngsoni* selectively consumed lycosids during our study period was obtained by comparing the raw frequency of occurrence of lycosids and other prey types in dunnart scats with the frequency of occurrence of the same prey taxa collected in invertebrate pitfall traps [29]. Lycosids also comprised 13% of all captures of prey by *S*. *youngsoni* that we witnessed directly [30]. Finally, we found lycosids to be preferred over other types of invertebrates in captive cafeteria-style experiments [29].

Based on the observations presented above, we hypothesized that lycosids are targeted by *S*. *youngsoni* due to their high energy or nutrient content compared with other prey types. Energetic and nutritional (protein and lipid) composition of different arthropod prey that are eaten by *S*. *youngsoni* was ascertained using a range of laboratory techniques. We tested the general hypothesis that prey composition influences selective predation by *S*. *youngsoni*, with the specific prediction that lycosids will have higher energy or nutritional composition than other prey types that are potentially available. We focused on quantifying the protein and lipid content of invertebrates for several reasons. Firstly, these two macronutrients form the greatest edible portion of arthropod bodies, and their concentrations vary substantially between species [31]. Additionally, both protein and lipid are important in prey selection by predators [7, 12, 31]. Carbohydrates were not quantified as these are generally found in relatively low concentrations in arthropod bodies [31, 32]. We do not report on body water composition of arthropods because our study was carried out during and after a period of high rainfall in the study region, thus providing no rationale to expect that lycosids would be selected due to their water content.

## Methods

Animal ethics approval was provided by the University of Sydney Animal Ethics Committee (Project Number: 2016/966). Research Ethics was not applicable. Appropriate permissions and licences to conduct the fieldwork were obtained from the Queensland Government (Permits WITK15192514 and WISP15192514).

### Study site

Study specimens were collected at Main Camp, Ethabuka Reserve (23°46’ S, 138°28’ E), in the north-eastern Simpson Desert, Queensland during five field trips in April, July and October 2016, and May and October 2017.

### Collection of arthropods

Specimens from several arthropod Orders–Araneae (including Lycosidae), Blattodea, Coleoptera, Orthoptera, and Scorpiones–were collected from the Main Camp site for compositional analysis. Only arthropods known to be eaten by dunnarts were sampled [27, 28, 33]. Arthropods were live-captured in vertebrate pitfall traps set on 16 permanent trapping grids (see [34] for details), or through opportunistic diurnal searches to capture grasshoppers and katydids (Caelifera and Tettigoniidae, respectively), using a hat as a net. Although *S*. *youngsoni* is nocturnal, diurnal arthropods were sampled because they are hunted by dunnarts at night while they are at rest or under cover [27]. Hand-sampling was also undertaken over several nights covering an area of ~3 ha around Main Camp, using a spotlight (Fenix TK35) to locate eyeshine [35, 36], and capture nocturnal arthropods such as arachnids (Lycosidae, Miturgidae). Captured arthropods were immediately placed into individual plastic vials or snap-lock bags to minimize water loss.

Given the absence of any detailed identification key for Simpson Desert arthropods [37, 38], collected specimens were identified to Order or Family (Coleoptera and Araneae). Each arthropod vial was labelled with a unique ID number to prevent misidentification, placed in a cool box out of direct sunlight, and as soon as logistically possible (maximum 6 hours; mean 2 hours), the collected arthropods were then placed into a cool room at ~3°C to slow their metabolism. The specimens were then transported on ice back to the University of Sydney and killed in a freezer. Samples were cooled rather than frozen to avoid the risk of accidental thawing and disintegration of specimens en route to Sydney.

#### Preparation of arthropod samples for energy and nutrient analyses

At the University of Sydney specimens were weighed to an accuracy of 0.0001 g before being dried for 72 h in an oven set to 60°C [39]. Following this, they were weighed a final time before being ground into a powder using a mortar and pestle to get samples as homogeneous as possible [26, 39]. As the lipid and protein analyses, and the determination of energy density, destroyed sample material, multiple individuals of the same family were pooled to generate sufficient material (at least 0.3 g; [39, 40]). Most families were represented by a single morphospecies, except for Lycosidae which had two morphs, and Carabidae, which had three morphs. However, preliminary analyses (energy) revealed no significant difference between the morphospecies of each family and so these were pooled together. This also follows the methods of [18] in which *S*. *youngsoni* prey selectivity was identified at a family level. The number of individuals needed for energy and nutrient analyses varied between invertebrate groups and samples. For example, one sample comprising a large scorpion needed only one other individual to yield the required mass, whereas another contained 10 miturgid spiders. Due to pooling of individuals, sample sizes were relatively small.

### Measurement of arthropod energy density

To determine the energy density of arthropod samples, we used a Phillipson Oxygen Microbomb Calorimeter (Gentry Instruments, Aiken, SC) coupled to a SP-G3C Speedex chart recorder (John Morris Scientific Pty Ltd; [41, 42]). Calorimetric assays require pellets weighing 0.01–0.02 g, so ~0.015 g (mean ± SE; 0.016 ± 0.0003 g) of dried sample was combusted in 100% oxygen [41]. This process was repeated twice per sample and averaged for increased accuracy. A benzoic ash standard was used to calibrate the calorimeter every 10^th^ sample and, after combustion, pellets were re-weighed to obtain the ash content and hence calculate the energy density in kilojoules per gram (kJ g^-1^), following [42], [39] and[41]. The known energy density of benzoic acid (i.e., 26.393 kJ g^-1^), was used to calculate kilojoules per millivolt (kJ mV^-1^) (1) and then kJ (2):

$$kJ\;{mV}^{-1}=ash\;free\;dry\;weight\;(g)\times 26.393\;kJ\;g^{-1}$$ (1)

$$kJ=\frac{kJ\;{mV}^{-1}}{mV}$$ (2)

The value from (2) could then be used to estimate kJ g^-1^ of invertebrate samples (3):

$$kJ\;g^{-1}=\frac{\Delta V\times kJ}{ash\;free\;dry\;weight\;(g)}$$ (3)

where *ash free dry weight* is the initial mass of the pellet minus the final mass of ash residue, and ∆V is the peak in voltage following combustion minus the initial voltage.

A single-factor analysis of variance (ANOVA) was performed to compare the energy densities between arthropod groups using R Version 3.3.3 [43]. Assumptions of normality and homogeneity of variance were checked and met using Shapiro-Wilks and Levene’s tests, respectively. Tukey’s HSD *post-hoc* test was used to distinguish which arthropod groups differed from each other. Energy values were expressed as kJ g^-1^ of ash free dry weight to avoid inaccuracy due to potential contamination by sand particles that stick to the arthropods and are difficult to remove [39].

### Analysis of lipid and protein content

Total lipids were extracted using a chloroform-methanol-water (1:1:1, by volume) mixture [41, 44, 45]. This method is described in full by [44], but in summary involved homogenizing the tissue in the chloroform-methanol-water mixture before centrifuging and separating the resulting pellet [46]. This process was repeated a second time and the lower chloroform phase was left to evaporate over 24–72 h before being re-weighed [46]. Total lipid content was calculated gravimetrically as the difference in mass before and after the extraction method was applied [10, 41]. This process was repeated three times for each sample, and these ‘triplicates’ were averaged to obtain a single value per sample.

Total arthropod protein content was ascertained via the Dumas combustion technique using a LECO FP628 (LECO, St. Joseph, MI) machine [41, 47]. Samples weighing 0.2 g were placed in the analyzer and combusted to produce carcass nitrogen content [10, 41, 48]. Crude protein was then calculated by multiplying the nitrogen value by a standard factor of 6.25 [10, 45].

Two separate single factor ANOVAs were employed to test whether lipid and protein content differed between arthropod groups. Assumptions of normality (Shapiro-Wilks) and homoscedasticity (Levene’s test) were tested, but even after applying square-root and arcsine transformations [49, 50], variances remained heteroscedastic and data non-normal for both lipid and protein. Hence, untransformed data were used in the final analyses and a more conservative α value (≤ 0.01) adopted for statistical significance. Additionally, we used the Dunnett T3 (1980) non-parametric *post-hoc* test for both lipid and protein data as this test is most appropriate when variances are unequal and group sizes are small (i.e., *n* < 50) and dissimilar [51, 52]. Tettigoniidae were omitted from protein analyses due to limited sample sizes (*n* = 2).

## Results

A total of 244 individual invertebrates were collected during the study, with a total biomass of 38.66 g (see S1 Table). Carabidae contributed the most with 64 individuals collected and a total mass of 12.73 g, while Tettigoniidae contributed the least with only 4 individuals collected and a total mass of 1.09 g.

### Energy

**Fig 1:**
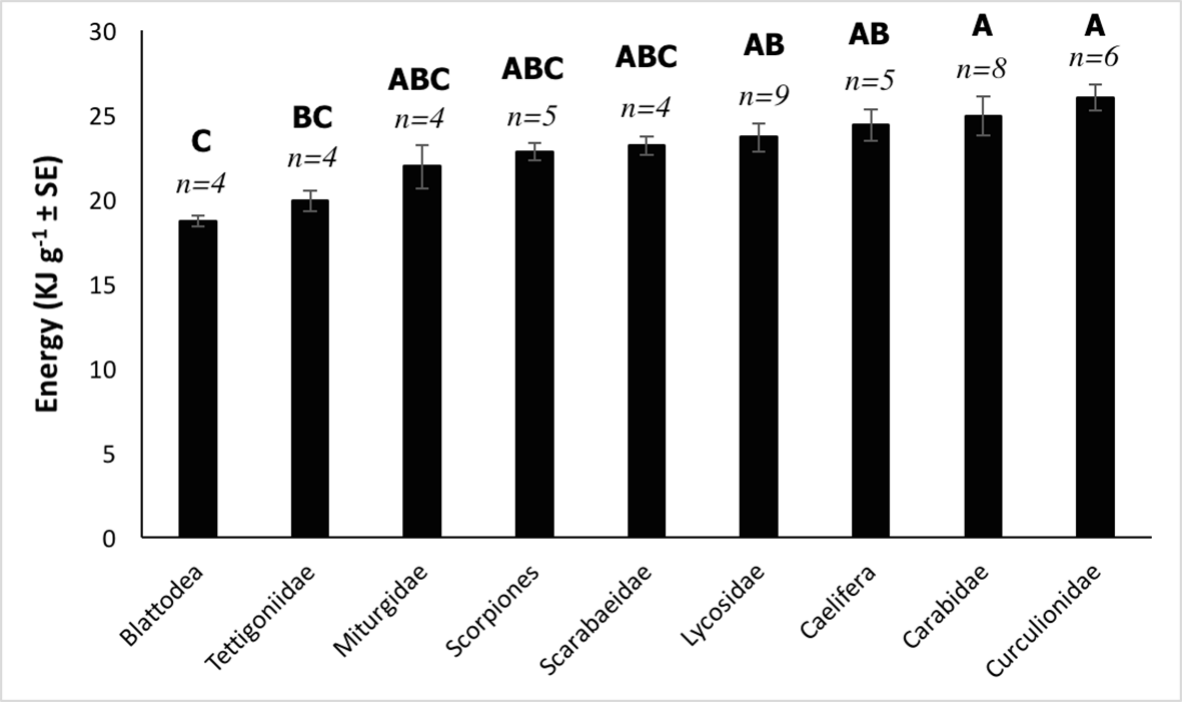
Mean energy content (kJ g^-1^ ± SE) of nine arthropod groups collected from the Simpson Desert, south-western Queensland, that occur in the diet of *Sminthopsis youngsoni*. Sample sizes (*n*) are shown above the bars. *Post-hoc* results for all groups are presented, with groups differing statistically from those with different letters.

Energy content differed significantly between arthropod groups (*F*_(8,40)_ = 5.51, *P* < 0.001), with weevils (Curculionidae) containing the highest energy density (26 kJ g^-1^) and cockroaches (Blattodea) the lowest (18.7 kJ g^-1^; Fig 1). *Post-hoc* tests revealed that Lycosidae did not have significantly higher energy compared to the other arthropod groups.

### Lipid and protein

**Fig 2:**
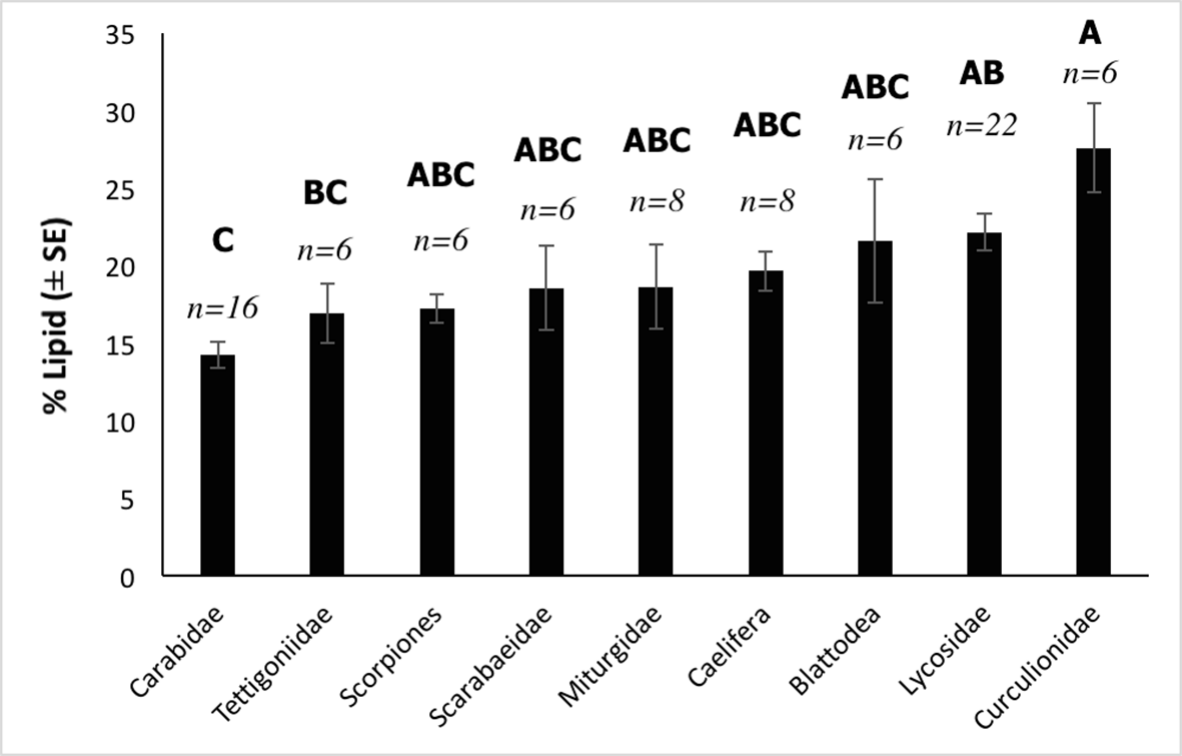
Total lipid content (% ± SE) of nine arthropod groups collected from the Simpson Desert, south-western Queensland, that occur in the diet of *Sminthopsis youngsoni*. Sample sizes (*n*) are shown above the bars. *Post-hoc* tests showed Curculionidae (A) to differ significantly from Carabidae (C).

**Fig 3:**
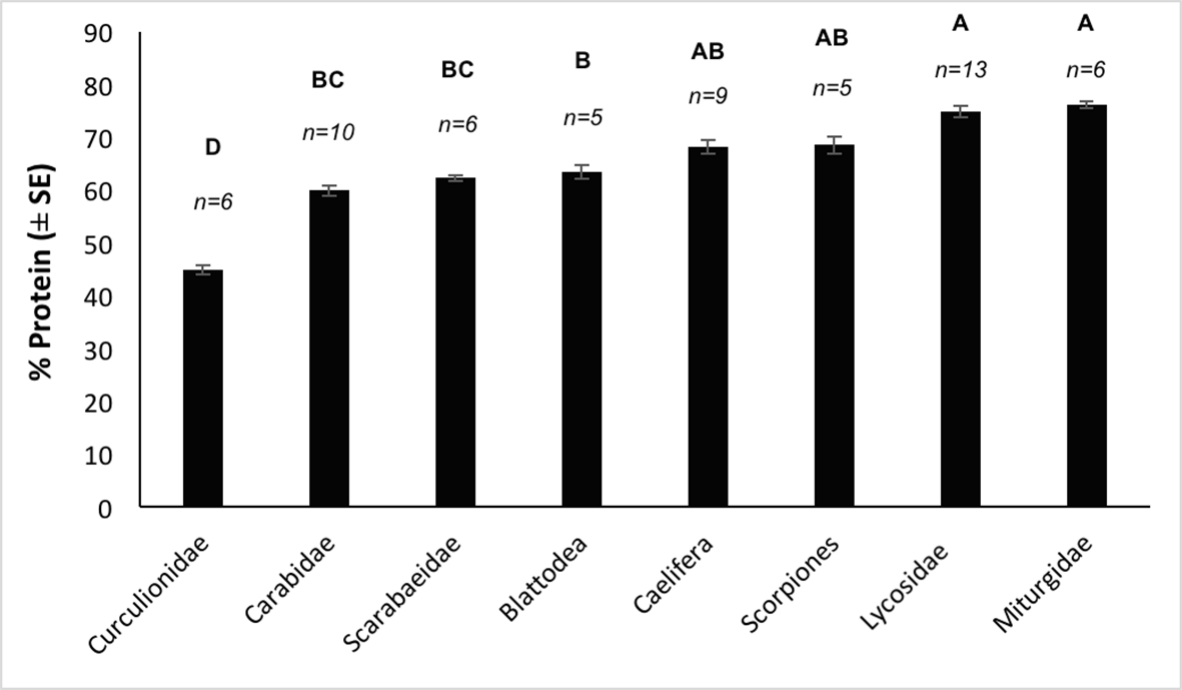
Mean protein composition (% ± SE) of arthropod groups collected from the Simpson Desert, south-western Queensland, that occur in the diet of *Sminthopsis youngsoni*. Sample sizes (*n*) are shown above the bars. *Post-hoc* results are presented for all groups, with different letters denoting a significant difference between groups. Tettigoniidae was omitted from analyses due to limited sample size (*n* = 2).

Lipid content differed between arthropod groups (*F*_(8,74)_ = 4.26, *P* < 0.001) with weevils (Curculionidae) containing a notably higher percentage of fat (27.6%) than all other groups (Fig 2). However, *post-hoc* tests revealed a significant difference only between Curculionidae and Carabidae. Protein content also differed significantly between invertebrate groups (*F*_(7,52)_ = 69.62, *P* < 0.001). Miturgidae and Lycosidae contained the most protein with 76.2% and 74.9%, respectively, while Curculionidae had a significantly lower protein content compared to all other groups (Fig 3).

## Discussion

Lycosids were found to contain relatively high proportions of energy, protein and lipid; however, these spiders did not stand out from other available arthropod groups in their yields of these food components. Although providing some support for the hypothesis that lycosids are depredated selectively by *S*. *youngsoni* because of their high energetic or macronutrient composition relative to other available arthropod prey, the high variance and overlaps in these food components between invertebrate groups suggest that alternative factors may also be at play. In the discussion below, we consider methodological and statistical limitations that may have contributed to our results, as well as alternative explanations.

Foraging theory has traditionally considered energy to be the primary focus for animals when foraging [2, 53, 54], with predators selecting prey that deliver the greatest net rate of energy gain [4–6]. Previous studies of insectivores have generally supported this concept, finding that animals employ energy-efficient foraging tactics by preferentially eating invertebrates that provide the greatest net energy yield [27, 55]. In contrast, results from the present investigation suggest that obtaining energy may not be a key priority for *S*. *youngsoni* when foraging; despite lycosids being selectively depredated, these spiders did not contain significantly more energy than other available prey. Lycosidae had a fairly high energy-density after Curculionidae, Carabidae and Caelifera, but did not contain significantly more than most other groups. Due to their hard exoskeleton, weevils may have longer capture and handling times, and therefore *S*. *youngsoni* may have to expend greater energy per unit gained compared to lycosids. Thus, the preference for *S*. *youngsoni* to select lycosids on the basis of energy content may be partly justified. However, grasshoppers (Caelifera) do not have a hard exoskeleton and have a similar energy-density to Lycosidae, indicating that overall, lycosids are not selected on the basis of their energy content alone.

It is possible that the methods we employed were not sensitive enough to detect real differences that existed in energy-density among the arthropod groups. However, this seems unlikely. Although sample sizes were not large (*n* = 4–9), samples were duplicated to increase accuracy, and standard methods were used at all stages of the assay procedure [41, 42]. In addition, the energy-density values obtained here are similar to those reported for the same arthropod groups in other works [26, 40].

Similar considerations can be brought to bear on the results for the total lipid and protein composition of the different arthropod groups. Curculionidae did appear to contain less protein and more lipid than other arthropods, with a significant difference found between Curculionidae and Carabidae in lipid composition (Fig 2) and all other groups in relation to protein composition (Fig 3). Assay methods for both protein and lipid followed standard protocols and returned results that were comparable to those reported in previous works [41, 44, 45]. Sample sizes are small; however, samples were based on triplicates (lipid) and duplicates (protein), with results averaged across these subsamples (as per [45]). Additionally, as samples were composed of multiple individuals, values are more representative of the group than if each sample comprised only one individual.

Miturgidae had the highest protein content followed closely by Lycosidae. This suggests that spiders (Araneae) in general may have high protein content but that lycosids *per se* do not stand out. Alternatively, it may be that *S*. *youngsoni* is selecting arthropods with the highest amount of ‘available protein’. Chitin contains a sizeable amount of bound protein, and thus the chitin concentration of arthropods may affect protein digestibility [56, 57]. As the hard exoskeletons of beetles contain large amounts of chitin it may be that they are less digestible compared to grasshoppers and spiders for instance, which have a higher proportion of digestible or available protein [57]. Hence, this may explain why *S*. *youngsoni* selects spiders over beetles. However, our earlier research has found that two other dunnart species are able to digest 70–85% protein from insect diets [45].

Taken together, these results provide little evidence that the sampled arthropods differ greatly from each other in terms of energy-density or macronutrient composition, or that lycosids differ markedly from the broader sample in any aspects of their body composition.

### Future directions

As the nutritional and energetic composition of invertebrates can vary with season and the body parts that are assayed [6, 7, 39], pooling entire individuals across trips may have masked subtle differences that existed in body composition between the arthropod groups. However, limited sample material meant that analyses at a finer resolution, for example between seasons, were not feasible. It would be beneficial to intensify sampling for invertebrates within seasons in future, and also to sample during wet and dry periods.

In addition, to comprehensively understand whether *S*. *youngsoni* forages to reach a specific nutritional state or intake target, a geometric framework approach could be employed in future studies. This framework takes into account multi-dimensional disparities in food composition, and fitness-related outcomes (e.g., reproduction, growth) can be measured and compared between individuals that reach these outcomes versus those that are restrained from doing so [7, 58]. Furthermore, compelling information regarding the nutritional drivers of foraging can be identified when an animal is forced to use the ‘rule of compromise’ [7, 59]. Specifically, when an animal is constrained to a diet with two nutrients in a ratio dissimilar to that of the target ratio, it is driven to make a decision to either over-ingest or under-ingest one nutrient to reach the target intake of the other, or to not reach the target for either nutrient [7]. Thus, specific nutritional or energy priorities can be ascertained. The disadvantage of testing the rule of compromise is that it must usually be trialled under artificial conditions in laboratory settings, thus reducing the potential applicability to animals foraging under natural conditions. To investigate invertebrate chitin concentration in relation to digestibility in more detail, in the future, digestibility studies would need to be undertaken. However, once again, to provide reliable and accurate data they need to be carried out in a captive situation to facilitate collection of total input and output (scats) material. Understanding prey digestibility may be important, as closely related fat-tailed and stripe-faced dunnarts (*Sminthopsis crassicaudata* and *S*. *macroura*, respectively) have been shown to optimise their intake by selectively eating body parts which are more digestible and discarding legs and antennae [45]. Alternatively, future studies could explore specific toxin, vitamin, amino and fatty acid, or micronutrient content of lycosids compared to other arthropod prey, as these factors may also shape foraging decisions [7].

Prey size and handling efficiency are also important considerations when investigating foraging behaviour as they relate directly to foraging theory and the prediction that predators should select prey that deliver the greatest energy per unit loss in effort or time [4, 27]. As such, it may be that *S*. *youngsoni* targets lycosids due to their ease of capture and short handling times relative to other invertebrate prey, such as weevils (Curculionidae). However, previous research involving *S*. *youngsoni* and other species of *Sminthopsis* has found no preference toward a particular prey size on the basis of handling time alone [60, 61]. There is also no evidence that other aspects of lycosid biology, such as their use of particular defensive behaviours or toxins, may render them easier to depredate than other prey; for example, once encountered dunnarts dispatch lycosids and highly venomous spiders such as some mygalomorphs with equal ease and speed [62]. Instead, other features of the predator’s environment, such as encounter rates or risk of predation and competition, may be more influential in shaping foraging selectivity [27].

## Conclusion

Investigation into the nutritional and energetic content of prey can provide valuable insight into the foraging decisions of predators and help to elucidate whether energy or nutrient-specific foraging is prioritized. Here, however, we found that lycosids contain no more energy or nutrients than do other potentially available arthropod prey, and hence that nutritional factors alone are unlikely to explain why *S*. *youngsoni* preys selectively on lycosids. It may be that alternative factors are more influential in shaping this selectivity. For instance, lycosids may have lower capture and handling times compared to other prey. However, as discussed previously, this is unlikely and selectivity is probably driven by factors in the environment of *S*. *youngsoni*. For example, selective depredation of lycosids may arise simply as a consequence of high encounter rates if *S*. *youngsoni* and lycosids occupy similar microhabitats and are active at similar times. By quantifying the spatial and temporal activity patterns, as well as diets of both groups of predator, the extent of resource overlap could be identified and alternative explanations for the observed pattern of selective depredation evaluated.

## Supporting information

S1 Table Invertebrate biomass. Biomass (as dry mass) of each taxonomic group along with the number of individuals of each group collected from the Simpson Desert, south-western Queensland.
(DOCX)
