# Supplementary material for: Understanding selective predation: Are energy and nutrients important?
Source: PLoS One. 2018 Aug 8;13(8):e0201300. doi: 10.1371/journal.pone.0201300 (PMC6082548; doi:10.1371/journal.pone.0201300)
Supplement: S1 Table — Biomass (as dry mass) of each taxonomic group along with the number of individuals of each group collected from the Simpson Desert, south-western Queensland. (DOCX) [file pone.0201300.s001.docx]

# Supporting Information

**Table 1.** Biomass (as dry mass) of each taxonomic group along with the number of individuals of each group collected from the Simpson Desert, south-western Queensland.

| **Invertebrate Group** | **Total Dry Mass (g)** | **Number of Individuals** |
| --- | --- | --- |
| Tettigoniidae | 1.09 | 4 |
| Blattodea | 1.86 | 16 |
| Miturgidae | 1.90 | 45 |
| Curculionidae | 2.52 | 10 |
| Scarabaeidae | 3.28 | 10 |
| Caelifera | 4.20 | 15 |
| Scorpiones | 4.67 | 19 |
| Lycosidae | 6.42 | 61 |
| Carabidae | 12.73 | 64 |
| **TOTAL** | **38.66** | **244** |
